# Supplementary figures and images for: Construction and validation of a novel prognostic signature of microRNAs in lung adenocarcinoma
Source: PeerJ. 2021 Jan 8;9:e10470. doi: 10.7717/peerj.10470 (PMC7798616; doi:10.7717/peerj.10470)

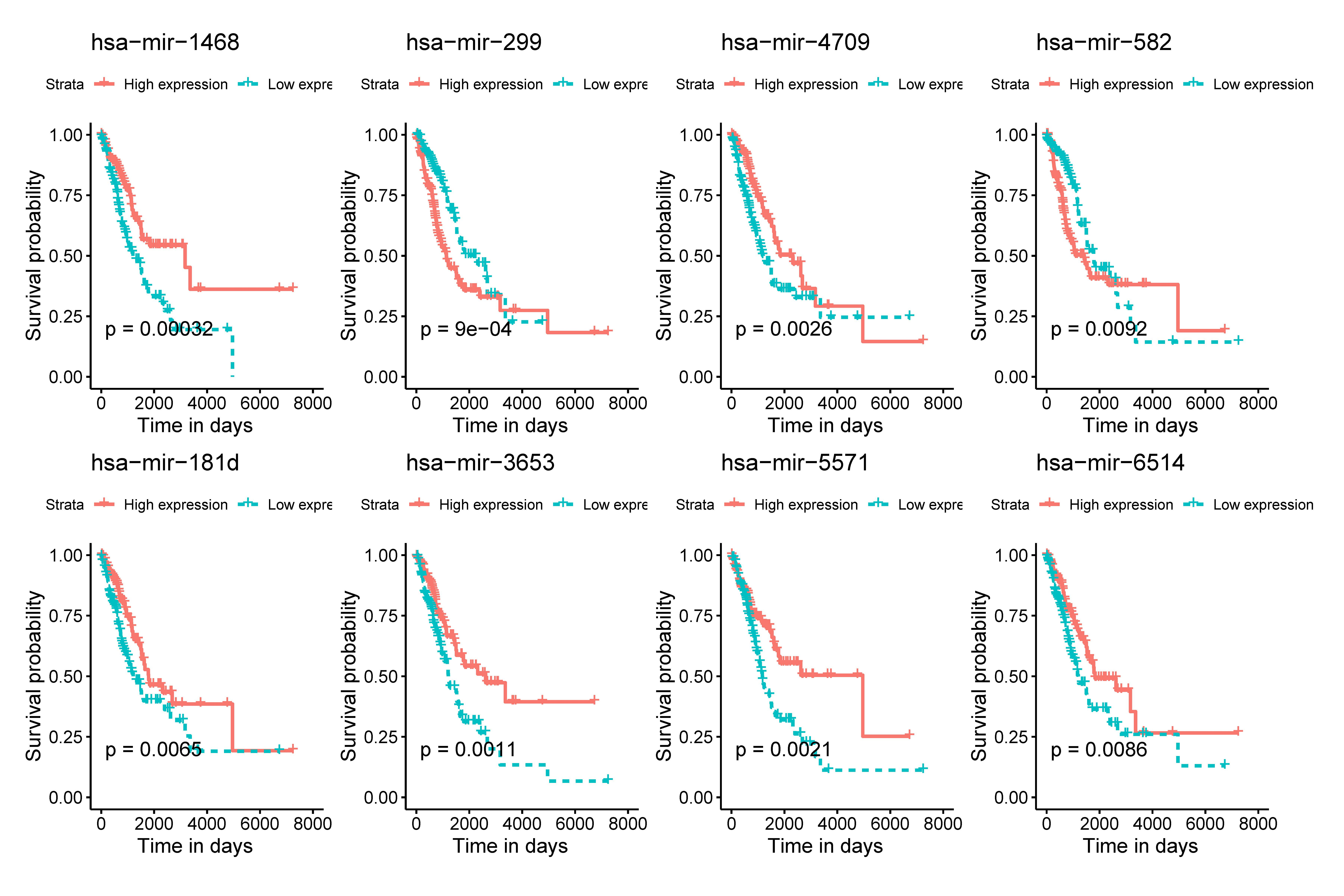

Supplement: Supplemental Information 3 [file peerj-09-10470-s003.png]

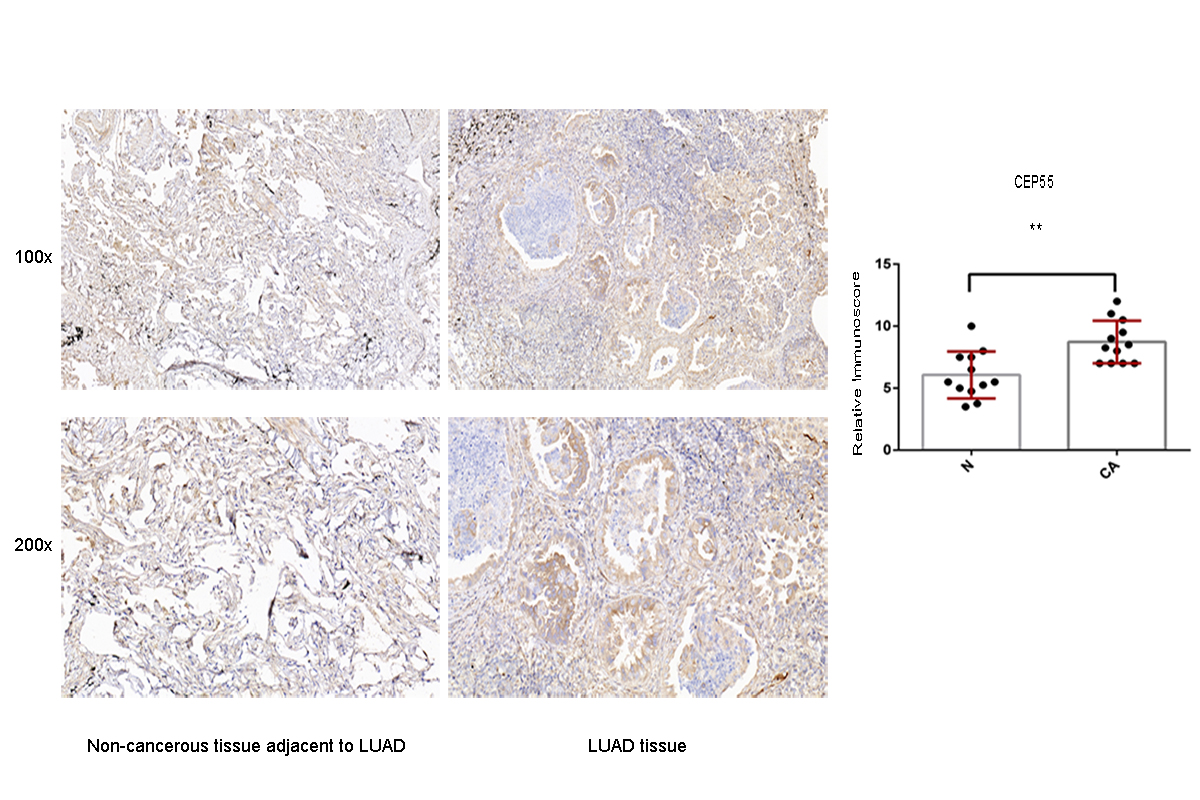

Supplement: Supplemental Information 4 — A value of P < 0.05 was regarded as statistically significant. ** P < 0.01. LUAD, lung adenocarcinoma. [file peerj-09-10470-s004.jpg]

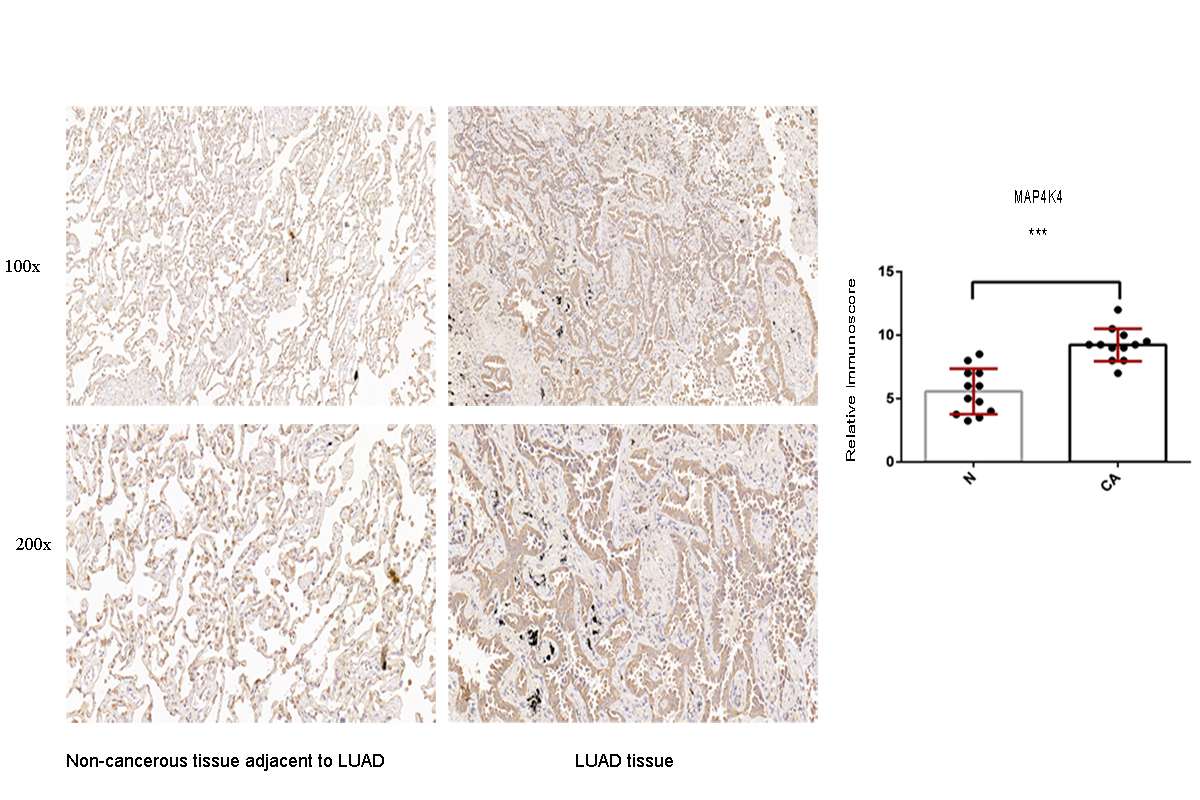

Supplement: Supplemental Information 5 — A value of P < 0.05 was regarded as statistically significant. *** P < 0.001. LUAD, lung adenocarcinoma. [file peerj-09-10470-s005.jpg]

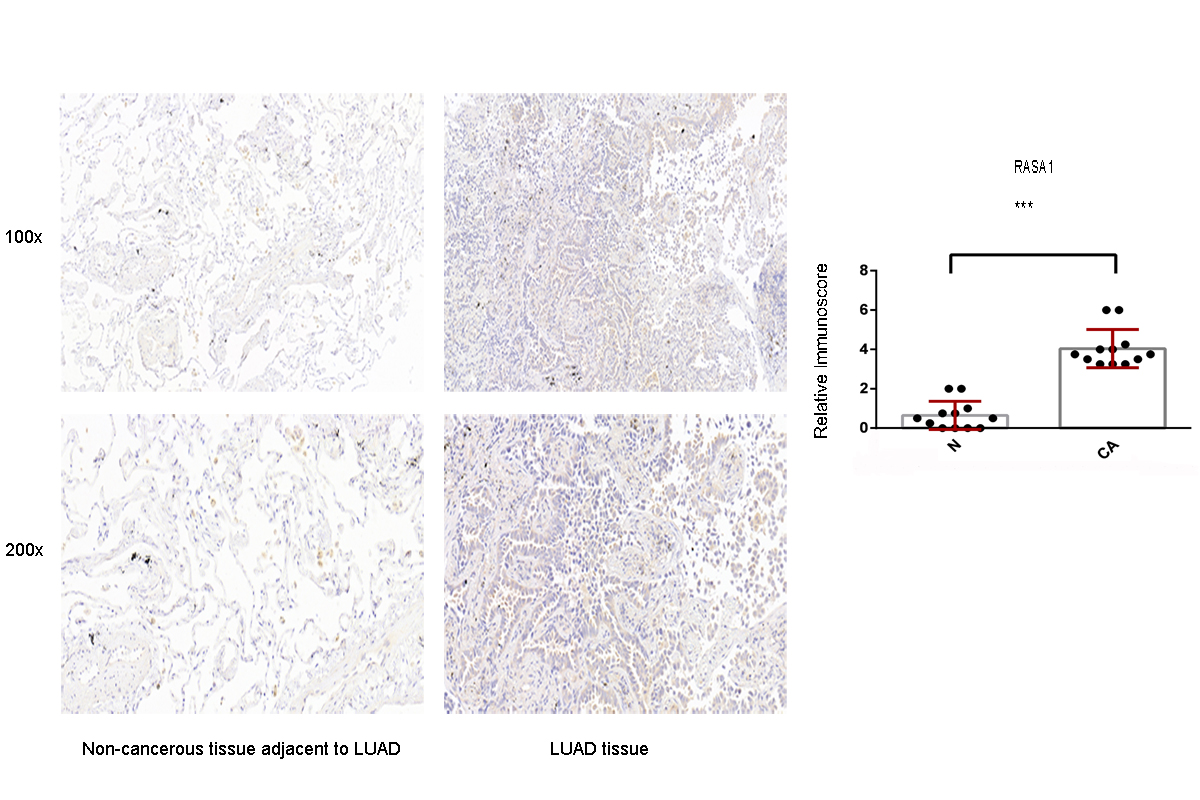

Supplement: Supplemental Information 6 — A value of P < 0.05 was regarded as statistically significant. *** P < 0.001. LUAD, lung adenocarcinoma. [file peerj-09-10470-s006.jpg]

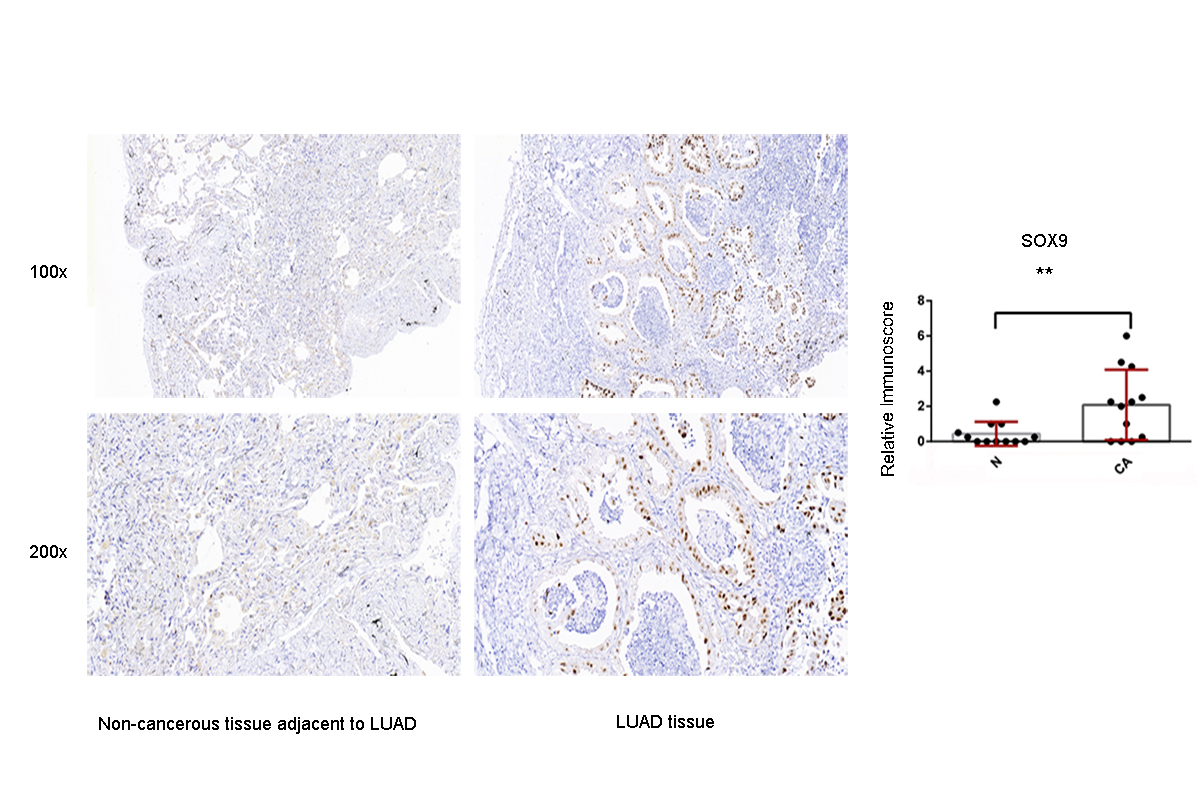

Supplement: Supplemental Information 7 — A value of P < 0.05 was regarded as statistically significant. ** P < 0.01. LUAD, lung adenocarcinoma. [file peerj-09-10470-s007.jpg]
